# Implementation and Effectiveness of Policies Adopted to Enable Breastfeeding in the Philippines Are Limited by Structural and Individual Barriers

**DOI:** 10.3390/ijerph191710938

**Published:** 2022-09-01

**Authors:** Jyn Allec R. Samaniego, Cherry C. Maramag, Mary Christine Castro, Paul Zambrano, Tuan T. Nguyen, Janice Datu-Sanguyo, Jennifer Cashin, Roger Mathisen, Amy Weissman

**Affiliations:** 1 Nutrition Center of the Philippines, Muntinlupa 1781, Philippines; 2 Alive & Thrive Southeast Asia, FHI 360, Quezon City 1101, Philippines; 3 Alive & Thrive Southeast Asia, FHI 360, Hanoi 11022, Vietnam; 4 Alive & Thrive Southeast Asia, FHI 360, Muntinlupa City 1770, Philippines; 5 Alive & Thrive Southeast Asia, FHI 360, Washington, DC 20009, USA; 6 Asia Pacific Regional Office, FHI 360, Bangkok 10330, Thailand

**Keywords:** breastfeeding policy, maternity protection, mother- and baby-friendly hospital initiative, mixed methods study, Philippines, the Code

## Abstract

The Philippines has adopted policies to protect, promote, and support breastfeeding on par with global standards, yet the impact of these policies is not well understood. This study assesses the adequacy and potential impact of breastfeeding policies, as well as the perceptions of stakeholders of their effectiveness and how to address implementation barriers. This mixed methods study entailed a desk review of policies and documents and in-depth interviews with 100 caregivers, employees, employers, health workers, and policymakers in the Greater Manila Area. Although the Philippines has a comprehensive breastfeeding policy framework, its effectiveness was limited by structural and individual barriers. Structural barriers included inconsistent breastfeeding promotion, limited access of mothers to skilled counseling, limited workplace breastfeeding support, gaps in legal provisions, weak monitoring and enforcement of the Philippine Milk Code, and the short duration and limited coverage of maternity leave. Individual barriers included knowledge and skills gaps, misconceptions, and low self-confidence among mothers due to insufficient support to address breastfeeding problems, misconceptions in the community that undermine breastfeeding, limited knowledge and skills of health workers, and insufficient support extended to mothers by household members. Breastfeeding policies in the Philippines are consistent with global standards, but actions to address structural and individual barriers are needed to enhance their effectiveness for improving breastfeeding practices.

## 1. Introduction

Despite the well-documented protection that breastfeeding provides to maternal and child health and survival, breastfeeding practices remain suboptimal worldwide [1,2]. The World Health Organization (WHO) and the United Nations Children’s Fund (UNICEF) recommend breastfeeding initiation within the first hour of birth, exclusive breastfeeding for the first 6 months of life, and continuous breastfeeding for up to 2 years and beyond [3]. According to the Global Breastfeeding Collective, a partnership formed by international agencies calling for increased investment in breastfeeding [4], 48% of children globally are breastfed within an hour of birth, 44% of children under six months of age are breastfed exclusively, and 68% and 44% of children still breastfeed at the ages 1 and 2 years, respectively [5]. In the Philippines, 74.0% of infants are breastfed within the first hour, and 57.9% of infants under six months of age are exclusively breastfed; at one and two years of age, 54.1% and 34.2% of children are still breastfeeding, respectively [6]. However, while the country’s rates of early and exclusive breastfeeding are above the global average, the prevalence of exclusive breastfeeding, continued breastfeeding at one year of age, and continued breastfeeding at two years of age remain below the 2030 Global Breastfeeding Collective targets of 70%, 80%, and 60%, respectively [5]. According to the World Bank, breastfeeding generates a significant economic gain of USD 35 for every dollar invested [7]. The global cost of not breastfeeding has been estimated at USD 341.3 billion annually, equivalent to 0.70% of the global gross national income (GNI) [8], and USD 3.8 billion in the Philippines, equivalent to 1.05% of the country’s GNI [9].

Although nearly all women are biologically capable of breastfeeding, the decision to breastfeed according to the recommendations is influenced by a variety of factors at the societal, community, household, and individual levels [1]. Interventions to improve breastfeeding outcomes can then be considered as addressing both structural (e.g., policy) and individual (e.g., knowledge and attitudes) determinants. According to the Global Breastfeeding Collective, there are seven policy actions that enable breastfeeding: (1) increasing funding to raise breastfeeding rates, (2) full implementation of the International Code of Marketing of Breast-Milk Substitutes and relevant World Health Assembly (WHA) resolutions (the Code), (3) enactment of paid family leave and workplace breastfeeding policies, (4) implementation of the Ten Steps to Successful Breastfeeding in maternity facilities, (5) improving access to skilled breastfeeding counseling, (6) strengthening links between health facilities and communities, and (7) strengthening of monitoring systems for policies, programs, and funding for breastfeeding [10]. The WHO also recommends that all pregnant women and mothers of young children receive at least six sessions of breastfeeding counseling within the continuum of maternal and childcare provided by trained healthcare professionals, community-based lay, or peer breastfeeding counselors [11].

In the Philippines, the Department of Health (DOH) has identified breastfeeding as a key component of national infant and young child feeding (IYCF) and maternal, neonatal, child health, and nutrition (MNCHN) policies. The DOH is the national government agency mandated to provide policy direction and develop plans, programs, and technical standards on health. It provides guidance to local government units (LGUs), which have the responsibility of delivering health services to the population. Programs being implemented in line with the IYCF and MNCHN policies include the essential intrapartum and newborn care program, which aims to increase the early initiation of breastfeeding, and the Breastfeeding TSEK (Tama, Sapat at Eksklusibo) to promote exclusive breastfeeding. The Philippines’ Rooming-In and Breastfeeding Act of 1992 provided incentives to health institutions for rooming-in and other supportive breastfeeding practices [12]. The Expanded Breastfeeding Promotion Act of 2009 mandated the establishment of lactation stations and paid lactation breaks in workplaces [13]. The Mother–Baby Friendly Hospital Initiative (MBFHI) accreditation program also established guidelines for providing an environment that promotes, protects, and supports breastfeeding during the maternity period [14]. Paid maternity leave, which is associated with improved breastfeeding practices [15], was also extended to 15 weeks through the enactment of the 105-Day Expanded Maternity Leave Law in 2019 [16].

Aggressive marketing and the widespread availability of breastmilk substitutes (BMSs) can influence social norms in favor of artificial feeding and undermine mothers’ confidence to breastfeed, resulting in lower breastfeeding rates [17]. The Code defines BMSs as any food being marketed or otherwise represented as a partial or total replacement for breastmilk, whether suitable for that purpose or not [18]. A BMS is a type of commercial milk formula (CMF) defined as artificial, ultra-processed products for infants and young children, including infant formula, follow-on formula, toddler or growing-up milks, formula for special medical purposes, and formula for pregnant and lactating women [19].

To limit inappropriate marketing practices and the harmful effects of the marketing of BMSs, feeding bottles, and teats, the WHA adopted the Code to guide Member States [18]. The Philippines was among the first countries to pass national legislation on the Code in 1986 [20], referred to as the Philippine Code of Marketing of Breastmilk Substitutes, or the Philippine Milk Code. In 2006, its Implementing Rules and Regulations were revised to provide detailed guidance for the implementation, monitoring, and enforcement of the Philippine Milk Code [21].

The Philippines’ CMF market is dominated by two transnational corporations, Nestlé and Reckitt Benckiser (Mead Johnson), with each holding 47% of the market share [9]. The CMF industry undermines breastfeeding through intensive marketing in the health system, widespread promotion of “growing-up” milks, and political strategies to weaken national policies that aim to protect breastfeeding [9].

While Philippine policies adopted to enable breastfeeding (e.g., Philippine Milk Code, Rooming-In and Breastfeeding Act of 1992, and Expanded Breastfeeding Promotion Act of 2009) conform to global standards, their impact is not yet understood. This study is conducted with the following objectives: (1) to assess the adequacy of the content of policies in place to protect, promote, and support breastfeeding in the Philippines in comparison with the seven policy actions identified by the Global Breastfeeding Collective; (2) to assess implementation, coverage, monitoring, and enforcement and to determine the potential impact of these polices; and (3) to document and examine the perceptions of caregivers, employees, employers, health workers, and policymakers in the Greater Manila Area of the effectiveness of breastfeeding policies and how to address implementation barriers.

## 2. Materials and Methods

### 2.1. Study Design

The data collection, analysis, and interpretation in this mixed methods study were guided by an existing conceptual model developed by Nguyen et al. [22] and entailed in-depth interviews (IDIs) and a desk review of relevant policies and documents.

### 2.2. Setting

The Philippines is a lower-middle-income country in southeast Asia [23] with a population of 109 million [24], with almost half of the population (47%) living in urban areas [25]. Each year, there are around 1.7 million live births [26]. The rates of neonatal mortality, infant mortality, and under-5 mortality are 14, 22, and 28 per one thousand live births, respectively [27]. In 2020, the gross national product (GNP) of the Philippines was worth USD 389.3 billion [28]. The country’s main sources of income are from services (60%) and industry (30%), as well as agriculture, forestry, and fishery (10%) [29]. Among southeast Asian countries, the Philippines has the highest overall ranking in the Gender Gap Index [30], but the country lags behind most of the countries in the region in terms of female labor participation rate [31]. It is only in recent years that the labor policies in the Philippines have closely aligned with global norms on maternity protection [32], with the enactment of the 105-Day Expanded Maternity Leave Act in 2019. This study was conducted in the Greater Manila Area, which includes the National Capital Region (NCR) and the neighboring provinces of Bulacan, Cavite, Laguna, and Rizal. These areas were selected because they are highly urbanized, have a high population density, include industrial zones and business districts with companies employing women, and have stable communication connectivity. The Greater Manila Area constitutes about 26% of the country’s total population [33], and the NCR alone contributes 32% to the country’s gross domestic product (GDP) [34]. The study sites are not representative of the Philippines as a whole, which remains predominantly agricultural outside of major urban areas. Given the COVID-19 prevention restrictions in place during data collection and the objective of collecting data from working women, the study sites were purposefully selected due to their feasibility, including considerations on internet connectivity to facilitate data collection.

### 2.3. Policy Review

Printed and electronic copies of policies, laws, survey reports, and program documents relevant to breastfeeding protection, promotion, and support were retrieved from national and local government offices and their official websites. The effective date, legal status, coverage, implementation strategies, and monitoring guidelines were extracted from these documents using a data extraction tool developed by the research team and compared with the International Code of Marketing of Breast-milk Substitutes and subsequent WHA resolutions, the WHO Baby Friendly Hospital Initiative, and International Labor Organization (ILO) Maternity Protection Convention, 2000 (Convention 183). The numbers and proportions of working mothers receiving maternity entitlements and details of monitoring and reporting activities were retrieved from relevant agencies, such as the Social Security System (SSS), the Civil Service Commission (CSC), and the Department of Health (DOH) or their official websites.

### 2.4. In-Depth Interviews among Stakeholders and Beneficiaries

#### 2.4.1. Sampling and Participants

Interview respondents consisted of key informants and community members who were selected through purposive sampling using the snowball technique.

For key informants, we invited 129 individuals, including 31 policymakers and implementers, 55 employers, and 42 health workers. The policymakers and implementers interviewed were identified by the research team and included representatives from national and subnational government agencies, non-government organizations, and development organizations that have roles in overseeing the development, implementation, monitoring, and enforcement of policies for the promotion, protection, and support of breastfeeding in the country. Employers were identified by regional program managers for nutrition, the national employers’ association, and personal contacts of the research team. Interviewed employers were either company heads, general managers, or human resource personnel of companies with at least 50 female workers. Interviewed health workers were either referred by key informants or local partners and worked in health facilities that provide maternal and child health and nutrition services. Of the initial 129 key informants identified, 44 agreed to be interviewed, and of them, 19 were policymakers and implementers, 13 were health workers, and 12 were employers (Table 1). A total of 44 interviews were completed with respondents who were predominantly women, and the interviews were mostly conducted through online platforms (Table 1).

For community respondents, we interviewed 56 individuals, including pregnant women, mothers, and their partners. The interviews were mostly conducted through online platforms (Table 1). The community respondents were selected through spot mapping, referral from local health centers, or author contacts within the study areas. Spot mapping involved visiting households in the communities to identify possible respondents. All respondents were at least 18 years old at the time of the interviews and provided written informed consent. Participant selection considered diversity in employment status (full time or part-time), work location (working from home, online, or in person), and current breastfeeding practices (breastfeeding, not breastfeeding, or mixed feeding).

#### 2.4.2. Data Collection

An informed consent form was emailed or handed to each eligible participant prior to the interview. All refusals were replaced with other candidates until the minimum number of respondents per category was met. In total, 100 participants were interviewed (Table 1). The interviews were administered in either Filipino, English, or a combination of both. Verbal consent for the interview and recording was audio-recorded at the time of the interview. In-depth interviews were conducted individually, either face-to-face (*n* = 47) or remotely using an internet-based video communication platform (*n* = 53), by a team of eight researchers. Ninety-five (95) participants agreed to audio recording. The research team recorded notes for the five interviews not audio-recorded. Participants who consented to the interview were assigned a personal identification number (PIN), which was linked to the data collected from each participant. Interviews were conducted between 3 December 2020 and 17 March 2021.

The major provisions on breastfeeding protection, promotion, and support policies present in the country were covered in the interviews. Policymaker participants were asked questions designed to elicit information on the following: their role in policy development, implementation, monitoring, and enforcement; their perception and experiences of the policy; and suggestions for improvement. Employers were asked about their implementation, monitoring, and enforcement of the policies in their workplace, as well as perceptions and suggestions for improvement. Pregnant women, mothers, and their partners were asked about their perceptions and experiences of the policies; suggested policy improvements; and sharing responsibilities of caring for children and domestic tasks.

#### 2.4.3. Data Analysis

For the assessment of policies, including regulations and strategies approved and instituted to protect breastfeeding (our first study objective), we categorized the data into provisions for promoting, protecting, and supporting breastfeeding. We then compared the contents of the Philippines’ government information sources with globally recommended policy provisions and regulations and identified gaps and discrepancies. Feedback on policies and regulations was also obtained through in-depth interviews. The interviews also provided data on the second study objective, i.e., to document the perceived effectiveness of policies and regulations and barriers against the optimal implementation and effectiveness of policies.

To analyze the in-depth interviews, audio recordings were uploaded to an internet-based storage drive accessible only to authorized team members and were transcribed in the language used during the interview. For those whose interviews could not be recorded, the interviewers took detailed notes of the interviews. The transcripts and notes were translated into English. NVivo software (v. 1.4.1, QSR International, Doncaster, Australia, 2021) was used to analyze the transcriptions and notes. Line-by-line coding was conducted until theoretical saturation was reached and emerging themes were identified. The transcripts and notes were coded independently by two members of the research team. The identified themes from the two coders were compared and discussed by the team and reviewed against data collected from the IDIs and desk review to produce more selective conceptual codes to explain larger segments of data.

The contents of the interviews were synthesized under the following themes that emerged from the analysis: adequacy of policy initiatives, effectiveness of the Philippine Milk Code, integration of breastfeeding interventions in health services, dissemination and monitoring of the Code, community and caregiver misconceptions about breastfeeding, gaps in addressing caregiver determinants of breastfeeding behaviors, and adequacy of public engagement through non-health systems.

## 3. Results

Our findings on the three study objectives are presented below. They reflect the themes identified in the analysis.

### 3.1. Policy Provisions for Promoting, Protecting, and Supporting Breastfeeding Are Consistent with Global Standards

Findings from the desk review and IDIs confirmed that the policy provisions to protect, promote, and support breastfeeding in the Philippines have expanded over time and are now more substantially aligned with global standards (Table 2, Appendix A).

While the DOH and the National Nutrition Council (NNC) are responsible for policy development, program planning, and support services to LGUs, the delivery and management of health services are under the jurisdiction of LGUs [35]. At the local level, midwives in government-managed health facilities provide services in the barangay (smallest political unit) and are heavily reliant on community health workers and barangay nutrition scholars [36]. The decentralized governance structure of the Philippines [37] means that policies that are adopted at the local level are optional. As of 2021, there were 43 local ordinances related to breastfeeding promotion and maternity protection retrieved from the NNC’s Compendium of Local Ordinances and Issuances [38], as summarized in Appendix B. The Kalusugan at Nutrisyon ng Mag-Nanay Act or Republic Act (RA) 11148 was enacted in 2018 to enforce and strengthen integrated strategies on IYCF and MNCHN implemented at the national and local levels [36]. The significance of the enactment of RA 11148 was affirmed in the IDI results.

*If you look at RA1148, it’s quite general, although it’s very specific on services that have health and nutritional services that have to be delivered. The IRR [implementing rules and regulations] made it a point to name agencies and tell them what to do. Even the members of the technical committee are given roles—specific roles on the IRR of RA1148. And that’s a standard that we should be putting into our own policies. Even RA1148 calls for a comprehensive sustainable strategy and manual of operations.* (Representative from national government agency, female, PIN 1002).

*Accountability defined in the law would be the DOH, and then the LGUs, [as] there is not much accountability for local government units. Whether they do it or not, there is no such accountability yet that is in place… Luckily, we have the RA 11148 that will help also in pushing for increased investments, as well as prioritization on the first one thousand days.* (Representative from national government agency, female, PIN 1018).

### 3.2. There Are Gaps in the Regulatory Framework to Protect Breastfeeding in the Philippines

The global status report of the WHO, UNICEF, and International Baby Foods Action Network (IBFAN) from 2020 assessed the Philippine Milk Code as “substantially aligned” with the International Milk Code (the Code) and gave its provisions perfect scores in terms of scope (20/20), monitoring and enforcement (10/10), and promotion in healthcare facilities (10/10) [39]. Its provisions on informational and education materials were mostly aligned with the Code but were given a score of 9/10, as there was no prohibition on referencing proprietary products in contents [39]. The provisions on engagement with health workers and systems scored 14/15, as sponsorships of fellowships, study tours, research grants, and attendance at professional conferences by manufacturers and distributors of BMSs were not prohibited, as long as they were disclosed to the health workers’ institutions, and donations of equipment and services were also allowed, as long as they did not refer to a propriety product [39]. The score on labeling was 12/15, and the report pointed the absence of provisions on recommended label requirements for follow-up formula on (1) the importance of breastfeeding for children 2 years and above, (2) the importance of exclusive breastfeeding for 6 months, (3) the recommended age for the introduction of the product, (4) prohibition on the use of image or text suggesting use by infants less than 6 months, and (5) prohibition of health professional endorsements [39]. The provisions on promotion to the general public were given a score of 10/20, as the Philippine Milk Code was assessed to have weak provisions prohibiting BMS manufacturers from advertising to the general public and contacting mothers [39].

### 3.3. Although Breastfeeding Support Is Integrated into Routine Health Services, There Are Implementation Gaps

Breastfeeding and IYCF counseling have already been integrated into routine health services in community-based primary care. There was also a referral system for postpartum women from hospitals to community settings as part of the MBFHI program. Home visitation and breastfeeding support groups were found to be active in the LGUs included in our study.

*Our breastfeeding promotion and home visits continue, according to instructions. Although our visitation only covered those with infants aged 6 months or below.* (Health worker, female, PIN 2006).

*Actually, for NCR (National Capital Region) we have more than 3000 breastfeeding support groups, and they have undergone this orientation on lactation management, and they are the ones giving counseling, preparing the mother and even at the time that the mother had encountered problems on breastfeeding.* (Health worker, female, PIN 2007).

Mothers’ responses indicated that the health system positively impacted their knowledge on the perceived protection and superiority of breastfeeding over artificial feeding.

*She [the mother] should try harder [regarding breastfeeding]. Because, from what I heard from the seminar, the first drop of breastmilk is good for immunity.* (Mother, PIN 5011).

Some respondents noted that not all health facilities and workers, especially community health workers, had sufficient understanding on breastfeeding messages and effective counseling skills and that pre-service training for health workers did not adequately cover breastfeeding.

*The health workers in the barangay [village], particularly the midwives, the barangay nutrition scholars, and the barangay health workers, are the least-trained or least-provided with formal training. These health care providers are the only source of information, that is why it is important that they give the right information.* (Representative from national government agency, female, PIN 1002).

Current breastfeeding promotion activities were perceived by some respondents to be small-scale and inconsistent compared to large-scale CMF marketing activities. Some respondents also pointed out that funding for breastfeeding promotion was not always available.

*But the biggest problem was, not all sectors understand their role in breastfeeding. That is one of the stumbling block(s). It is not included in the priorities of the LGUs, thus, it will not be funded.* (Representative from national government agency, female, PIN 1002).

*For funding, it is really an LGU decision. If the local chief executives are convinced of the importance of the activity, then the local health nutrition workers are given the resources.* (Representative from national government agency, female, PIN 1018).

Despite breastfeeding recommendations from health workers, there were gaps in breastfeeding support because of insufficient knowledge, skills, motivation, and time to address mothers’ breastfeeding challenges. The high turnover and workload of the health workforce also limited opportunities for health workers to attend training seminars, practice skills, and provide counseling services. Some suggested strengthening the technical capacity and service delivery system to breastfeeding support groups that can focus on providing practical support for mothers’ breastfeeding difficulties.

*I delivered my baby in that facility, and the midwife will just tell me, just practice breastfeeding.* (Mother who had difficulty breastfeeding, PIN 5015).

*We only have a few health workers, and no one can tangibly support mothers on their lactation problems. Usually, 1 or 3 days of training in counseling is not enough. You need practice, as you will be facing a lot of situations to test your skills. And in support groups, you can discuss these situations. And all of them will scale their skills on counseling and will give support to the mothers. There should be an agency to anchor for that.* (Representative from national government agency, female, PIN 1002).

Respondents reported that, while health workers know the superiority of breastmilk either from the mother or human milk banks over formula feeding, they do not make the effort to assess and correct breastfeeding problems and easily resort to prescribing CMF when challenges arise. In facilities with human milk banks, a lack of support for breastfeeding can reportedly contribute to over-reliance on pasteurized donor human milk, contrary to national guidelines.

*For example, when they have the impression that the mother has insufficient milk, which is not necessarily correct, they quickly resort to prescribing infant formula. And the sadder development was, yes, they will push for breastfeeding at the start, but will prescribe pasteurized donor breastmilk for various reasons. And it has become a routine. This is very sad, because the mother still doesn’t need to supplement, but they already lose confidence.* (Breastfeeding counselor and coordinator, female, PIN 2008).

### 3.4. Gaps in Monitoring, Inaccurate Communication, and Inadequate Enforcement Reduce the Effectiveness of the Milk Code

The DOH reported that the online platform for the reporting of Philippine Milk Code violations resulted in 474 reported violations from 2013–2020 [40]. The adoption of a crowdsourced monitoring platform in 2018 increased the number of reports [41]. In 2020, there was a significant increase in reported violations on CMF donations because of rampant donations during post-disaster events and the COVID-19 pandemic [42].

The crowdsourced Code monitoring system includes a website and a mobile application. However, the level of awareness of the population on the Philippine Milk Code and access to the online platform has limited its effectiveness. As mentioned by stakeholders and health workers, the vagueness of policy provisions can make it difficult to recognize actual violations. Respondents also revealed that current monitoring mechanisms and the roles and responsibilities of different agencies were unclear, with monitoring at the local level found to be lacking. Marketing tactics such as CMF industry sponsorship and involvement in conferences, seminars, and sponsored recreation trips were difficult to document and penalize. In addition, the unclear definition of what constituted a violation and the lack of legal support weakened the prosecution of reported violators.

*Many understand that the Milk Code only applies to infants, but with the revised IRR, anything that will undermine breastfeeding, for example, would be considered a violation, so there is still a lack or limited understanding of the Milk Code.* (Representative from national government agency, female, PIN 1018).

*The problem is we do not know how to handle it [on seminars and conferences with BMS involvement]. There is no procedure, such as how to gather information on the events and incentives, especially when attendees and companies will not tell us. On that part, it would be cumbersome, especially if we have no lawyer for case building or assisting us. Personally, I cannot point out what is the violation that is specifically stated in the Milk Code, as not all violations are written in the policy, and are subject to interpretation.* (Regulator, female, PIN 1029).

*We’re asking for task forces that are not present in all regions and even provinces, municipalities, especially on monitoring sponsorships and donations. We’re quite passive about it.* (Programmer, male, PIN 1001).

Respondents also believed that the current sanctions for violators were too light, especially for the CMF industry. Violators of the Philippine Milk Code are punishable by imprisonment of two months to 1 year, or a fine that ranges from PHP 1,000 to PHP 30,000 (USD 20 to USD 600), or both [20].

*The sanctions given are very low, for example, for doctors, the individual [fine] are only up to PHP 30,000.* (Breastfeeding counselor and coordinator, female, PIN 2008).

### 3.5. Loopholes in Maternity Leave and Lactation Policies in Workplaces Limit Mothers on Continuing Breastfeeding

Most working mothers also perceived that they needed to use CMF when they returned to work. Since 2019, mothers are entitled to longer maternity leave with the enactment of the 105-Day Expanded Maternity Leave Law, which extended the paid maternity leave duration from the previous 60–78 days to 105 days (3.5 months) [16]. The law made the paid maternity leave duration in the country on par with the minimum recommended maternity leave duration set in Convention 183 [43]. However, despite this recent extension of the length of leave, the 3.5 months leave entitlement still falls short compared to the recommended 6 months of exclusive breastfeeding.

Mothers who were informally employed, such as those under contract employment, were also not eligible to receive maternity entitlements. Some respondents reported not receiving any cash entitlements during leave.

*I am just new here, under Job Order (JO), so I don’t have these. They only provided me 3 months [of maternity leave], but it does not have maternity pay. So, I don’t have a salary [when I was on maternity leave].* (Mother, PIN 5028).

Interviews with female employees also revealed that some employers used short-term contracts to avoid the provision of maternity entitlements to employees.

*From what I heard in the factory, they don’t pay contributions for the benefits. Because I know in the factory, contracts end every 5–6 months, after which they will have to be renewed again.* (Pregnant woman, PIN 4011).

Most mothers were aware of how to express and store breastmilk; however, only a few mentioned the use of lactation stations and lactation breaks in workplaces. Mothers viewed breastmilk expression in the workplace as inconvenient, impractical, and unattainable. Certain types of work also required more time and stress management and, thus, posed greater difficulty for mothers to continue breastfeeding upon returning to work. Some also mentioned the lack of necessary equipment for expression and storage as a barrier to continuing breastfeeding.

*For mothers who need to work, it is usually difficult to pump and pump, then you still have to carry it.* (Mother, PIN 5007).

*There are ways to continue breastfeeding, you will just need perseverance. The equipment needed to express and store breastmilk is also expensive. You also need to monitor the expressed milk because this must be put in the freezer. This has a timeframe on its usage, or else it will be spoiled. So, the mother must really be dedicated to conduct this practice.* (Mother, PIN 5004).

### 3.6. Some Mothers Reported Barriers and Were Not Confident about Recommended Breastfeeding Practices

Health workers mentioned that the most common difficulty of mothers during breastfeeding was when they felt that they did not produce sufficient breastmilk. According to interviews with mothers and health workers, infant feeding cues such as crying led mothers to doubt their breastmilk supply. Mothers reported feeling worried that their child might not receive enough nutrition through breastfeeding. A few of them expressed confusion and frustration, as they knew the advantages of breastfeeding, but they did not know what to do when faced with breastfeeding difficulties.

*My baby will always cry every time she breastfeed…. I was able to produce breastmilk, but it is very little.* (Mother, not breastfeeding 5-month-old child, PIN 5025).

*I really don’t produce breastmilk. I don’t know if it is because formula milk was already given to her in the hospital… I pity my child, she just cries and cries.* (19-year-old mother, not breastfeeding 2-week-old child, PIN 5008).

*I don’t know what to do, what if I don’t have enough supply? That is why you will be confused; will you still force breastfeeding, or will go to infant formula instead.* (Pregnant woman with formula-fed young child, PIN 4008).

Mothers commonly mentioned the following misconceptions related to breastfeeding: not all mothers have the capacity to breastfeed, the ability to breastfeed is associated with breast size and body frame, and mothers who practice breastfeeding have poor financial status and an unpresentable appearance. These beliefs were mentioned to justify the use of CMF.

*It really varies. There are moms who really don’t have breastmilk, while there are moms who have many. It will depend on the mother’s body frame.* (Mother, PIN 5027).

### 3.7. Some Mothers Used CMF despite High Awareness on the Protection Conferred by Breastfeeding

Nearly all mothers were aware of the positive impact of breastfeeding on health and household economic status, and some mentioned that non-breastfed infants were more likely to get sick compared to breastfed infants. Few mothers knew about the additional importance related to cognitive function and nutrition. Even those who were unable to breastfeed or preferred not to breastfeed appeared to have a good understanding of the protection that breastfeeding provides mothers and children.

Most of the interviewed mothers mentioned health workers’ advice, as well as disclaimers at the end of CMF commercials and on labels of CMF products, as sources of information on the superiority of breastmilk over CMF.

*But you can also see it on the packaging of the formula milk, it is written there that the formula milk is only a substitute for breastmilk, because breastmilk is still the best for babies.* (Mother, PIN 5027).

But despite this knowledge, mothers were easily influenced by the promised benefits of CMF because of difficulty in balancing time between work and breastfeeding, as well as lactation difficulties. These problems could be rooted from the structural gaps, as discussed beforehand.

*In my opinion, because she [the mother] is a busy person, that is why she wanted to try what was endorsed in the commercial, thinking that ‘Oh, this is good, maybe I will try this formula milk first instead of breastmilk’.* (Pregnant mother, PIN 4011).

*In my experience, I had difficulty expressing breastmilk for three days, no breastmilk came out. Thus, someone said to me that I should feed my baby infant formula to fill his stomach.* (Mother, PIN 5010).

The prohibition of CMF products in health facilities and CMF donations were perceived negatively by some respondents, especially restrictions on donations during emergencies.

*It [bottle feeding] is prohibited in the hospital. So, after we got out of the hospital, we shifted to bottle feeding.* (Mother, PIN 5018).

*They will say [that] we are already under the emergency (COVID-19 pandemic), yet we are still being strict with the Milk Code*. (Health worker, female, PIN 2004).

*In health centers, feeding bottles are really prohibited. Conflicts often exist since some mothers and caregivers, especially the elderly, will really insist on bringing those.* (Health worker, female, PIN 2004).

### 3.8. There Is a Need to Promote and Support Breastfeeding outside the Health System: In Workplaces, Households, and Communities

Our study also revealed the importance of extending breastfeeding promotion within the workplace, household, and community to provide a supportive breastfeeding environment and improve breastfeeding behaviors. Insufficient knowledge and low motivation to provide proper breastfeeding support seemed to result in the underutilization of lactation stations.

*We conducted a survey; they have provided space for the breastfeeding station, but no advocacy activities are being conducted on the benefits of breastfeeding and how it will be done. The HR personnel do not know how long the storage is and how the milk will be transported if they are far from work. We need to invest in teaching mothers how to do it [breastmilk expression and storage]. Do they need a cooler when transporting it? They want to breastfeed, but they do not know how to do it.* (Civil society advocate, female, PIN 1028).

Findings also suggested that household members were important for ensuring the continuity of support for breastfeeding mothers at home. Fathers who received breastfeeding counseling mentioned giving verbal support, providing foods and supplements that were perceived to increase breastmilk supply, assisting with breastmilk storage and expression, and helping with household chores to allow the mother to focus on breastfeeding. On the other hand, a father’s insufficient knowledge resulted in worries and confusion as they observed their partners struggling with breastfeeding. This type of father tended to accept the use of CMF when faced with challenges.

*We really don’t know anything, when our child cries, we think that he or she is hungry. Although others said that bottle-feeding might overfeed the baby.* (Father, PIN 6009).

*People need to continue working for a living. Those [infant formula] will really be needed.* (Father, PIN 6026).

Table 3 summarizes the identified barriers at the individual and structural levels that limited the implementation and effectiveness of polices to enable breastfeeding.

## 4. Discussion

This study represents the first comprehensive review of the present status of national policy initiatives to protect, promote, and support breastfeeding in the Philippines. Our first and second objectives were to assess the adequacy of the contents of these policies compared with the seven policy actions identified by the Global Breastfeeding Collective, as well as to assess the implementation, coverage, monitoring, enforcement, and potential impacts of these policies. The findings of this review showed that national policy initiatives were largely aligned and consistent with global recommendations. However, some provisions lacked clarity (e.g., definitions of Code violations) and specific details that undermined their full implementation. Some provisions only evolved in the past few years (e.g., extension of paid maternity leave), so their full implementation and impact may not yet be evident among stakeholders and implementers.

Our third objective was to identify barriers that impeded the implementation and effectiveness of the policy initiatives in terms of improving breastfeeding practices. Our study showed that most mothers understood the importance of breastfeeding for child health and development, but that this understanding did not necessarily translate into good breastfeeding practices. This finding is consistent with previous studies, such as the Philippines National Nutrition Survey in 2015, in which 89.8% of mothers reported intending to breastfeed their child at birth during their current or last pregnancy [44]. However, in 2019, only 66.5% believed that breastmilk was the best milk for the baby, and the intention to breastfeed exclusively in the Philippines was 57.9% [6], while by 6 months of age, only 35.1% of children were exclusively breastfed in the Philippines [6]. Wood et al. reported that mothers had trouble applying their knowledge and skills when they encountered actual breastfeeding difficulties after birth [45]. A study commissioned by the NNC showed that the most common reasons given by mothers to stop breastfeeding included the perception of insufficient breastmilk, breastfeeding discomfort as a result of poor latching and positioning, and the eventual return to work outside the home [46]. Excessive crying of the infant [47], inconvenience, and fatigue [48,49] were also reasons given by mothers to discontinue breastfeeding. This suggests that the provision of breastfeeding information is insufficient and must be combined with skilled breastfeeding counseling and psycho-social support to address the underlying determinants, requiring the large-scale engagement of communities, family members, peers, co-workers, employers, and health systems [50].

The crying of infants and the inability to settle infants while breastfeeding leads to a subjective perception of insufficient breastmilk, which results in the unwarranted use of BMSs [47]. The perception of insufficient breastmilk is linked to a lack in maternal confidence in the ability to breastfeed since most mothers depend on the child’s hunger cues to measure their milk supply [51,52]. Without knowledge of how to self-assess breastmilk adequacy and increase their supply, mothers are left unsupported and feel inadequate. The findings of a systematic review were in accordance with the self-efficacy theory, wherein a mother’s confidence in her ability to breastfeed predicted her breastfeeding behavior [50]. Evidence also suggested that effective intervention strategies should include a multilevel approach since an individual’s knowledge, behavior, and attitudes were influenced by interactions between friends, family, and the community [50,53].

Overcoming individual barriers requires support to address perceptions of low milk supply and increase self-efficacy to breastfeed through evidence-based counseling that includes assessment of breastfeeding status [54], self-assessment of breastmilk supply, how to increase breastmilk supply, interpretation of infant behavior, and proper latching and positioning [45]. Breastfeeding support groups and peer counseling to provide practical support on breastfeeding difficulties would likely improve self-efficacy and exclusive breastfeeding [50,55,56]. Counseling should be provided and made available both before and after birth, rather than in one period only [47,50], and should include family members to create a supportive environment and proper assistance for the lactating mother. The lack of paternal attendance in breastfeeding classes [57] and the lack of support from family members can lead to the discontinuation of exclusive breastfeeding [58,59]. The vacuum created by the lack of support is filled by aggressive marketing practices, including misinformation that questions the adequacy of breastmilk and idealizes substitutes instead [19].

We know that policy action must result in sufficient scale-up and the adequate coverage of services and programs to bring changes in behavior [60]. Both national and local policies were required to incentivize or mandate expectant mothers to give birth in health facilities, and these actions contributed to an improvement in the institutional birth rate in the Philippines, from about 50% in 2010 to 89% in 2020 [38,61,62]. The inadequate scale and coverage of breastfeeding initiatives are also key issues. While implementation of the MBFHI policy in health facilities increased breastfeeding initiation rates and breastfeeding duration [50,63,64], coverage remains insufficient. As of 2020, only 41% of health facilities in the Philippines were MBFHI-accredited, and no birth centers were included [42]. There is also a need for the continuous capacity-building of health workers on technical knowledge and counseling skills, as well as continuous reminders to ensure the delivery of consistent information and the provision of effective support before problems occur and when mothers encounter breastfeeding challenges. The IYCF program assessment report recommended strengthening the IYCF training of educators and students under allied health courses and integrating IYCF into the basic education curriculum [36]. In addition to universal counseling, referral systems for specialized counseling services on lactation and mother support groups must be set up and made accessible through the use of local and online contacts and continuous promotion in digital media. Strengthening the capacity for providing breastfeeding support with quality and high coverage through the healthcare system can reduce CMF supplementation to infants in hospitals, which delays the early initiation of breastfeeding and negatively affects exclusive breastfeeding and breastfeeding duration [65,66] and can also prevent over-reliance on donor human milk in hospitals with human milk banks [67].

Program implementers must strengthen breastfeeding promotion and assistance within workplaces [1,48]. The Philippines’ Expanded Breastfeeding Promotion Act encouraged but did not require the partnering of workplaces with local health offices for technical assistance when setting up lactation stations [13]. Employers must be educated and supported to create awareness and appreciation of maternity protection policies and to proactively conduct breastfeeding advocacy among workers [68].

The findings of the study also identified the short duration of maternity leave entitlement and the limited paid maternity leave coverage of those who were informally employed. Note that the 15 weeks of maternity leave entitlement for Filipino mothers is just one week more than the minimum recommended leave duration based on ILO Convention 183 [43]. Increasing paid maternity leave to a minimum of 6 months would make the leave duration consistent with the recommended 6 months of exclusive breastfeeding, enabled by the proximity of the mother and infant. A publicly financed cash transfer program to improve the coverage of maternity entitlements to informally employed women was investigated [69].

Despite the strength of BMS marketing regulations, mothers in our study were routinely exposed to BMS marketing through circumventions of the national code, such as cross-promotion and digital marketing. A study in the NCR in 2021 revealed a recent proliferation of BMS promotion through digital channels. Here, 44% (*n* = 145) of mothers saw at least one BMS promotion within the previous 6 months, mostly from TV advertisements [70]. The CMF industry uses tactics to circumvent restrictive policies, such as supporting breastfeeding activities and partnering with health and humanitarian programs to gain credibility and promote products [71]. The presence of CMF representatives in health facilities is an issue in other southeast Asian countries as well, including Vietnam [72]. By using similar packaging and brand names across products, the CMF industry creates associations between claims for “growing-up” milks with infant formula in the same product line [9,73]. Another study in the Philippines in 2011 showed that formula feeding was significantly associated with the recall of formula advertisements and receiving recommendations to use formula milk from doctors [74]. Our study also recorded negative perceptions of the public on the strict regulation of BMS, especially during emergencies. This might be due to low motivation and limited knowledge on the adverse effects of artificial feeding. Evidence from the region has suggested that investments in large-scale multimedia breastfeeding promotions would improve breastfeeding behaviors and the social acceptance of breastfeeding [75], such as the use of large-scale TV, radio, and digital media campaigns [76,77]. The Philippine IYCF Strategic Plan 2030 recommended the development of a communication strategy through existing accessible communication platforms but, to date, is not yet fully implemented [36].

The IYCF strategic plan of 2011–2016 targeted an increase in MBFHI accreditation, the creation of IYCF support groups, workplaces with lactation stations and lactation breaks, the implementation of sanctions to Milk Code violators, the inclusion of IYCF curricula in elementary to tertiary schools, and compliance on infant feeding in emergencies [78]. However, the country has lagged behind its targets [36]. Our study showed gaps due to the unclear definition of violations, difficulty in monitoring all types of violations, lack of legal assistance, limited resources, lack of local-level implementation, and the light sanctions imposed on violators. The 2018, the Philippine IYCF program assessment reported that violations were promptly investigated, but the imposition of sanctions was delayed [36]. There were no actions on the observed conflicts of interest of health workers and professional organizations engaging with the CMF industry for conference sponsorships and research initiatives in violation of national laws. The establishment of a clear and comprehensive system for reporting of violations, especially at the local level, was recommended [36] to aid the current monitoring procedures. The legal gaps in the current regulations on BMS marketing should also be addressed and coupled with measures to increase awareness of their scope, prohibitions, and other implementing rules.

In order to reach the 70%, 80%, and 60% Global Breastfeeding Collective targets for exclusive breastfeeding, continued breastfeeding at one year of age, and continued breastfeeding at two years, respectively, the Philippines needs to address the individual and structural barriers to breastfeeding identified in this study. Interventions that were successful in other countries to address these same challenges were to strengthen breastfeeding policies as follows: (1) improved political commitment and leadership with stricter regulations, clearer definitions, and rigorous enforcement; (2) systematic monitoring and reporting backed with local regulations; and (3) prosecution of violators from multinational industries [79]. This should include an intensive health promotion component to address and correct, among others, misconceptions that contradict recommended breastfeeding practices, such as those linking capacity to breastfeed to physical features (e.g., body frame) and the perception of breastfeeding as an indicator of lower socioeconomic status despite knowledge of the economic impact of breastfeeding on household economics.

We acknowledge several limitations to our study, including the purposive sampling design, the inclusion of author contacts in the study, and the limited area coverage, which may affect the generalizability of the data. The study may not reflect the situation and perception of stakeholders, employers, health workers, and mothers outside the Greater Manila Area. However, given that national program managers in government and non-government agencies were included in the study, the study likely obtained broader national insights.

## 5. Conclusions

Although most policies adopted to enable breastfeeding in the Philippines are consistent with global standards, barriers to recommended breastfeeding practices remain. These include limited support from health workers and household members to address breastfeeding difficulties, as well as from employers upon return to work. Implementation gaps undermine the effectiveness of policies adopted to enable breastfeeding, including inconsistent breastfeeding messaging and support, conflicts of interest in the health system, lack of investments for human resources needed for adequate implementation and promotional activities, misunderstanding of the scope and provisions of policies to enable breastfeeding, and lack of enforcement of existing policies and regulations. These barriers may be addressed by increasing national financing and advocacy for additional local funding for breastfeeding promotion, widely adopting prohibitions on marketing by the CMF industry in the health system, and developing a service delivery network between health facilities and community-based and online breastfeeding support groups for providing practical support for all pregnant women and mothers. The Philippine example sheds light on the challenges and necessary actions beyond the adoption of policies, giving it an opportunity to continue to be a “lighthouse country”, not only for policy adoption but also for implementation.

## Figures and Tables

**Table 1:** Number of in-depth interview participants by gender and data collection method.

Type	Total	Female	Online
Policymakers and implementers	19	15	15
Health workers	13	13	13
Employers	12	11	11
Pregnant women	12	12	12
Mothers of infants aged 0–5 months	13	13	11
Mothers of infants aged 6–11 months	16	16	12
Partners of women with child aged below 12 months	15	0	13
TOTAL	100	80	87

**Table 2:** History of key policy provisions on breastfeeding protection, promotion, and support in the Philippines.

Key Provisions	1986–1991	1992–2005	2006–2010	2011–2017	2018–Present
Breastfeeding protection					
Information and education: provision of objective and consistent information on IYCF; availability of materials on IYCF for pregnant women and mothers	✓	✓	✓ (refined mechanisms)	✓	✓
General public: prohibition of 1) advertising and other forms of promotion to the general public of products within the scope of the Philippine Milk Code; 2) distribution of samples, special displays, discount coupons, premiums, special sales, and gifts; and 3) marketing personnel contact with pregnant women and mothers	✓	✓	✓ (refined mechanisms; issued guidelines for donations)	✓	✓
Healthcare system: requirement to promote and protect breastfeeding through policies and regulations; prohibition of BMS promotion, BMS company representatives, and display of BMS names and company logos	✓	✓	✓ (refined mechanisms)	✓	✓
Health workers: defining roles on encouraging and protecting breastfeeding; prohibition of receiving financial or material inducements from BMS manufacturers; prohibition of distributing samples of BMSs; required disclosure of benefits received from BMS companies, including fellowships, study tours, research grants, and attendance at professional conferences	✓	✓	✓ (refined mechanisms)	✓	✓
Manufacturer or persons employed by manufacturers and distributors: prohibition of involvement in any breastfeeding promotion activity; sales volume of BMS products not to be included in the calculation of incentives such as bonuses	✓	✓	✓ (refined mechanisms)	✓	✓
Labeling requirement: include information on the superiority of breastmilk, appropriate use of the product, potential harms of using the product, instructions on product preparation, and advice that product use should be only upon recommendation of health workers	✓	✓	✓ (refined mechanisms; issued guidelines on labeling BMSs)	✓	✓
Quality product: should meet the standards recommended by the Codex Alimentarius Commission and the Codex Code of Hygienic Practice for Foods for Infants and Children	✓	✓	✓	✓	✓
Monitoring and enforcement: issuance of guidelines for monitoring of violations and issuance of sanctions			✓ (issued sanctions)	✓ (issued guidelines for monitoring BMS activities)	✓
Breastfeeding promotion					
Promotion in healthcare system: breastfeeding promotion in health institutions; issuance of guidelines and incentives for health institutions adopting MBFHI		✓ (1992)	✓	✓	✓
Promotion in general public: information dissemination on breastfeeding among mothers and public; organization of breastfeeding groups in the community		✓ (1992)	✓	✓	✓
Promotion in workplace: requirements to have workplace policies on breastfeeding promotion and breastfeeding entitlement among workers; requirement to establish lactation spaces and provide lactation breaks				✓	✓
Integration in school curriculum: inclusion of breastfeeding key messages in curriculum of health-related schools				✓	✓
Breastfeeding support					
Rooming-in and breastfeeding in health facilities: issuance of guidelines and incentives for adopting rooming-in and breastfeeding support, including training of health workers on lactation management and infant care, maintaining rooming-in facility, and practicing prescribed methods for infant deliveries		✓ (voluntary)	✓	✓	✓
Adoption of Mother–Baby Friendly Hospital Initiative: issuance of guidelines on implementing the Ten Steps to Successful Breastfeeding adopted from UNICEF and WHO global criteria		✓ (voluntary)	✓ (revitalized; voluntary but required for PhilHealth-accredited facilities)	✓ (voluntary)	✓ (voluntary)
Adoption of essential newborn care protocol: issuance of policies and guidelines for health facilities to adopt essential newborn care protocol				✓	✓
Human milk bank: encouraging the establishment of human milk banks in health institutions				✓ (voluntary)	✓ (voluntary)
Paid maternity leave: provision of maternity leave entitlement and mechanisms for mothers to receive payment while on leave	45 days	60–78 days with pay (1997)	✓	✓	105 days with pay plus optional 30 days without pay (2019)
Adoption of Mother–Baby Friendly Workplaces: issuance of guidelines for mother–baby friendly workplace certification; recommendations for breastfeeding support and promotion in the workplace				✓ (voluntary)	✓ (voluntary)
Infant and Young Child Feeding: strategic plan identifying goals, targets, strategies, programs, and projects for IYCF				✓	✓
Local-level enforcement of breastfeeding policies: encouraging LGUs to create local policies supportive of breastfeeding					✓

✓ Check marks represent inclusion of identified key provisions in policies within the indicated time frames.

**Table 3:** Structural and individual barriers that limited the implementation and effectiveness of policies to enable breastfeeding.

Structural Barriers	Individual Barriers
Health System ▪ Limited availability of skilled counseling support Workplaces and communities ▪ Limited access to skilled counseling support Societal Level ▪ Inadequate breastfeeding promotion activities targeting communities, workplaces, and households ▪ Gaps in legal provisions, inadequate communication, and weak monitoring and enforcement of the Philippine Milk Code, which facilitates inappropriate marketing of commercial milk formula, including cross-promotion and engagement of industry representatives with health professionals ▪ Duration of maternity leave entitlement inconsistent with the recommended 6 months of exclusive breastfeeding, and insufficient mechanisms to ensure maternity protection coverage for the informally employed	Mothers ▪ Inability to address early breastfeeding problems, misconceptions that undermine breastfeeding, and insufficient knowledge on how to continue breastfeeding while working Fathers ▪ Insufficient knowledge on how to support breastfeeding mothers Health workers ▪ Insufficient knowledge, skills, motivation, and time to address mothers’ breastfeeding challenges

## Data Availability

Not applicable.

## Appendix A

**Table A1:** Summary of Philippine policies and provisions related to breastfeeding protection, promotion, and support.

Philippine Policy	Policy Coverage	Main Provisions
Breastfeeding Protection		
Date Published: 20 October 1986 Executive Order 51 “Adopting a National Code of Marketing of Breastmilk Substitutes (BMS), Breastmilk Supplements and Related Products, Penalizing Violations thereof, and for other Purposes” (also known as the Philippine Milk Code)	Information and Education	- Objective and consistent information is provided on infant and young child feeding - Informational and educational materials dealing with the feeding of infants and intended to reach pregnant women and mothers of infants and young children is required to include:(1) benefits and superiority of breastfeeding, (2) preparation and maintenance of breastfeeding, (3) negative effect of partial bottle-feeding, (4) difficulty of reversing the decision not to breastfeed, (5) proper use of infant formula along with the social and financial implications of its use, and (6) health hazards of improper use of infant formula
	General Public and Mothers	- Advertisements, promotions, and other marketing materials, including donations of products under the scope of the Code, is prohibited, unless approved by an Inter-Agency Committee (IAC) tasked with reviewing marketing materials within the scope of the Code and identifying those responsible for monitoring compliance to the Code - Special displays, discount coupons, premiums, special sales, loss-leaders, and tie-in sales (at retail level) shall not be allowed, but does not include price lowering on a long-term basis - Provision of gifts and utensils that may promote the use of BMSs or bottle feeding either directly or indirectly is prohibited
	Healthcare systems	- Breastfeeding promotion and protection policies and regulations: Ministry of Health shall take appropriate measures and provide objective and consistent information, training, and advice to health workers; health education classes shall emphasize hazards and risks of improper use of BMSs - No promotion of BMSs: no healthcare facilities shall be used for promotion of infant formula and other products under the scope of the Code and shall preclude dissemination of information provided by BMS manufacturers - BMS representatives: professional service representatives or similar personnel provided or paid by BMS manufacturers or distributors shall not be permitted in the healthcare system
	Health Workers	- Should encourage and protect breastfeeding - Use of information provided by BMS companies: information provided is only restricted to scientific and factual matters and shall not imply superiority to breastfeeding - Financial or material inducements to promote BMS: shall not be offered by BMS manufacturers to health workers or members of families and shall not be accepted by health workers and members of families - Provision of BMS samples, equipment, and utensils: shall not be provided to health workers and no health workers shall give samples of infant formula - Assistance for research and continuing education from BMS manufacturers: BMS companies may assist in research, scholarships, and continuing education of health professionals in accordance with the rules and regulations of the Ministry of Health
	Persons employed by manufacturers and distributors	Personnel employed in marketing of products within the scope of the Code shall not perform educational functions in relation to pregnant women or mothers of infants
	Labeling	Labeling requirement for products within the scope of the Code: (1) information about appropriate use of the product, (2) superiority of breastmilk, (3) products should be used with consultation of health workers, (4) instruction on preparation and potential harms, (5) shall not have pictures of texts which may idealize use of infant formula, (6) food products that do not meet requirements of infant formula shall carry label warning, (7) shall conform with rules and regulations set by the government
	Quality	Products within the scope of the code should meet applicable standards recommended by Codex Alimentarius Commission and the Codex Code of Hygienic Practice for Foods for Infants and Children
	Enforcement and Monitoring	- The established IAC is tasked with reviewing and examining all advertising, promotion, and other marketing materials within the scope of the Code, as well as identifying who is responsible for monitoring compliance - IAC given authority to (1) delete or prohibit objectionable portions of advertising media and other marketing materials and (2) to cause prosecution of violators of the Code
Date Published: 07 May 2004 DOH Administrative Order No. 18 s. 1997 “Labeling of infant formula ‘breastmilk supplement’ or ‘follow-on formula’	Labeling	Issued under the Philippine Milk Code, amendment to require use of terms “breastmilk supplement “or “not a breastmilk substitute” on labeling follow-on formula or breastmilk substitutes
Date Published: 15 May 2006 DOH Administrative Order 2006-0012 Revised Implementing Rules and Regulations of Executive Order No. 51, otherwise known as “the Milk Code”, relevant International Agreement Penalizing Violations Thereof, and for other Purposes		Defined mechanisms of implementation for the Philippine Milk Code, clarified coverage of the code, and defined penalties for violations
	Enforcement and Monitoring	- Department of Health (DOH): Primarily responsible for monitoring of the Milk Code and IRR; in coordination with other agencies, should (1) adopt monitoring guidelines for national, regional, and provincial levels; (2) provide regular training on monitoring compliance and enforcement; and (3) form monitoring teams at national, regional, provincial, city, municipal, and barangay levels - Monitoring teams at regional and provincial levels shall monitor, file, investigate, and resolve problems and violations arising at this local level. Violations that require prosecution shall be elevated to the Food and Drugs Administration (FDA) - Monitoring teams to submit regular reports on status of implementation to FDA - FDA shall investigate and verify reports of violations, apply administrative sanctions, and file criminal complaints for confirmed violators
Date Published: 28 May 2007 DOH Administrative Order 2007-0017 Guidelines on the Acceptance and Processing of Foreign and Local Donations During Emergency and Disaster Situations	General Public and Mothers	Defined the policy parameters and systematic management of donations for emergency and disaster situations, including prohibition of infant formula, breastmilk substitutes, feeding bottles, artificial nipples, and teats
	Monitoring and Enforcement	- Health Emergency Management Bureau (HEMB) of the DOH: develop procedures for monitoring local donations - Bureau of International Health Cooperation (BIHC) of the DOH: develop procedures for monitoring foreign donations - FDA: review and evaluate donated items within jurisdiction and issue a report - Centers for Health Development (CHDs) of the DOH: oversee distribution and utilization and submit a utilization report
Date Published: 01 December 2009 DOH Department Circular 2009-0228 Guidelines for the Monitoring of Milk Code Activities	Enforcement and Monitoring	- Provision of guidelines for monitoring and compliance to the Philippine Milk Code and its IRR at national, regional, and provincial levels - Provide composition of monitoring teams at the national, regional, provincial, city, municipal, barangay levels and define functions - DOH shall provide continuing education to monitoring teams, as well as provide reporting forms and mechanics - Monitoring teams are encouraged to request assistance to civil societies and non-government agencies for monitoring - Monitoring teams are furnished by FDA with information related to all applied events, sponsorships, research, and advertisements for monitoring - Monitoring teams are instructed to submit regular reports on status of Milk Code implementation - FDA should provide immediate feedback (within 5 working days) on actions taken based on the received reports of alleged violations
Date Published: 01 December 2009 DOH Department Circular 2010-0147 Guidelines for Physicians on the Promotion, Protection, and Support of Breastfeeding	Healthcare System and Workers	- Reiterate and increase awareness of physicians on breastfeeding and their responsibilities for the health of infants and young children - Emphasize extent of laws and ensure compliance with Philippine Milk Code - Physicians should be equipped with proper skills in infant and young child feeding (IYCF) and be part of routine in regular prenatal, delivery, and post-natal care
Date Published: 23 March 2020 NNC Nutrition Cluster Advisory No1, Series 2020 Nutrition Cluster Guidelines on LGU Nutrition Actions Relative to COVID-19	General Public and Mothers	- Reiteration of the provisions of the Philippine Milk Code on donations for local government unit (LGU) nutrition actions - Specific precautions on distribution of milk supplements for 3-year-olds and above are provided
	Healthcare System	- Reiteration of the Philippine Milk Code on health management for LGU nutrition actions - Promotion and support of breastfeeding for target mothers and children during emergencies
Date Published: 15 May 2020 DOH Department Memorandum 2020-0231 Guidelines on the Standardized Regulation of Donations, Related to the Executive Order 51, series of 1986, to Health Facilities and Workers, Local Government Units, Non-Government Organizations, and Private Groups, and Individuals in Support to the Response to Emergencies, Disasters, and Situations Where Health and Nutrition of Mothers, Infants, and Young Children are Affected	Information and Education	- Banning of branding and display of milk products on donations and related activities
	General Public and Mothers	- Specific precautions on distribution of milk supplements for 3-year-olds and above are provided; only the Secretary of Health and the IAC are authorized to request or approve donations covered by scope of the Philippine Milk Code. - DOH CHDs with the regional offices of the National Nutrition Council (NNC) shall lead families, support groups, and health workers to ensure practices that promote breastfeeding
	Healthcare System	- DOH CHDs with the regional offices of the NNC shall lead families, support groups, and health workers to ensure practices that promote breastfeeding- Reiteration that the use of artificial feeding shall only be considered after all means of breastmilk feeding have been exhausted
	BMS company staff	- Prohibited from using donations as part of their marketing campaigns - Shall not hold activities under the guise of classes, seminars, and recreation activities
	Labeling	- Packaging of milk donations must not bear branding and logo, but label should contain clear, legible instructions on how to prepare the product
	Quality	- No milk products with remaining shelf life less than 3 months may be donated
	Enforcement and Monitoring	- DOH CHDs with NNC regional officers to monitor donations and IYCF information and educational activities
Date Published: 22 May 2020 DOH Department Circular 2020-0217 Reiteration of the DOH Department Memorandum 2020-023	General Public and Mothers	Clarification on the regulation of donations to general and public health system:(1) ensuring that the safest, most sustainable means of feeding, breastfeeding, and lactation support are in place in local government units; (2) provision of proper nutrition education to recipients of products covered by Section 3 of the Philippine Milk Code; and (3) guidance on public relations and communications related to the distribution of these commodities
Breastfeeding Promotion		
Date Published: 02 June 1992 Republic Act 7600 “An Act Providing Incentives to all Government and Private Health Institutions with Rooming-In and Breastfeeding Practices and for other Purposes”	General Public and Mothers	- Information dissemination for pregnant women during prenatal, perinatal, and postnatal consultations and organization of breastfeeding support groups - Information, education, and communication (IEC) materials on maternal and infant care in health institutions
	Healthcare System	- Issuance of guidelines and incentives for private and government health institutions adopting rooming-in and breastfeeding, which include continuously teaching and training women on lactation management and infant care, maintaining rooming-in facility, and practicing prescribed methods for infant deliveries
	Health Workers	DOH with the assistance of other agencies, professionals, and assistance of non-government organizations shall conduct continuing information, education, and training programs for health workers on current and updated lactation management
	Enforcement and Monitoring	- Periodic monitoring and evaluation of IYCF strategy integrated into DOH monitoring coaching team and regular hospital assessment system - Incentive and award system: NNC to give regular awarding integrating IYCF indicators - Continued research and development for improving feeding practices
Date Published: 16 March 2010 Republic Act No. 10028 Expanded Breastfeeding Promotion Act of 2009		Amendment of Republic Act 7600: included requirements on establishment of lactation stations and lactation breaks, inclusion of breastfeeding in school curriculum, encouragement to establish breast milk banks in health institutions
Date Published: 16 Sep2011 DOH Department Circular 2011-0365 Guidelines for the “Mother–Baby Friendly Workplace Certification”		- Issued guidelines for application and qualification for certification on ‘working mother–baby friendly workplace’ - LGU to review and assess application; applications are endorsed by DOH CHDs for granting certifications, awarding, or resolution; random validation may be conducted
Infant and Young Child Feeding		
Date Published: 23 May 2005 DOH Administrative Order 2005-0014 National Policies on IYCF	Healthcare System	- A guide adapted from Global Strategy for IYCF for health workers and other concerned parties, which included (1) feeding in exceptionally difficult circumstances and (2) feeding options for HIV-positive mothers- Support systems: access to skilled IYCF support from health facilities and community-based networks, sustenance of Mother–Baby Friendly Hospital Initiatives, reiteration of Republic Act 7600 and Philippine Milk Code
		- Designation of committees and coordinators for IYCF management at national, regional, and provincial levels- Designation of IYCF interagency group for technical assistance
Date Published: 26 March 2018 Department of Interior and Local Government (DILG) Memorandum Circular 2018-42 Adoption and Implementation of the Philippine Plan of Action for Nutrition (PPAN) 2017–2022		- Enjoined LGUs to formulate local nutrition action plans (LNAPs) and include in their local development plans (LDPs) and annual investment programs (AIPs) to ensure adequate funding - Issued guidelines on roles and responsibilities of DILG Field Offices and LGUs in the implementation of the PPAN 2017–2022
	Enforcement and Monitoring	- DOH together with NNC and other agencies to develop monitoring and evaluation plans - NNC to establish a system for monitoring and maintain database on functional local nutrition committees - Established role of NNC and DILG of monitoring semestral report of LGUs on nutrition action programs
Date Published: 29 November 2018 Republic Act 11148 An Act of Scaling Up the National and Local Health and Nutrition Programs Through a Strengthened Integrated Strategy for Maternal, Neonatal, Child Health, and Nutrition (MNCHN) in the First One Thousand (1000) Days of Life, Appropriating Funds Therefore and for Other Purposes, otherwise known as the “Kalusugan at Nutrisyon ng Mag-Nanay Act”		- To provide comprehensive, sustainable, multisectoral strategies and approaches for MNCHN programs with active participation of DOH, NNC, national government agencies (NGAs), LGUs, civil society organizations and private sector - Strengthen and define roles of DOH, NNC, and other government agencies involved in implementation of the First 1000 Days - Strengthen enforcement of the Philippine Milk Code and Republic Act 10028
Date Published: 29 May 2019 DOH Memorandum Circular 2019-0027 Implementing Rules and Regulations of Republic Act (RA) No. 11148	General Public and Mothers	- DILG supported by local resolution shall ensure implementation and integration of the program and advocacy in LNAPs and training participation - Barangay nutrition scholars, barangay health workers, and community health workers should be mobilized and granted benefits, including PhilHealth - Designation of NNC technical committee - NNC to provide incentives and award system
	Enforcement and Monitoring	DOH, in coordination with NNC, Department of Agriculture (DA), LGUs, and other NGAs concerned shall be responsible for implementation - DOH and NNC should plan and conduct joint monitoring and review of programs and review incentives and award system; NGAs and LGUs to submit reports to NNC and DILG - LGUs are encouraged to create ordinances in relation to the Act, which may include enforcement
Date Published: 21 October 2019 DILG and DOH Joint Memorandum Circular No. 2019-0001 Guidelines on the Integration of Specific Programs, Projects, and Activities (PPAs) from the Philippine Plan of Action for Nutrition (PPAN) 2017–2022 to the Local Development Plans, Investment Programs, and Budget of Local Government Units.	Adoption of Policies for LGUs	- Issued prescribed guidelines on the integration of specific PPAs from PPAN 2017–2022 to LDPs, and provided specific guidance for allocation of local funds for nutrition in LDPs and corresponding LDPs and AIPs - NNC (1) to pursue efforts to strengthen the capacity of DILG Field Offices to assist in LGU compliance to law and (2) to provide technical assistance in integration of nutrition concerns through conducting local nutrition-planning workshops, development, orientations on nutrition planning, etc., with special emphasis on identified areas with significant nutritionally vulnerable populations - Coverage: All provincial governors, mayors of highly urbanized cities, municipal mayors, punong barangays, members of Sanggunian Panlalawigan, Panlungsod, Bayan, Barangay, DILG regional, provincial, city directors, and others concerned - Funding - Local funds may be tapped for nutrition PPAs; LGUs shall also consider NGAs (DOH, Department of Social Welfare and Development, Department of Education) complementary PPAs
	Enforcement and Monitoring	- LGUs: review their latest accomplishments in relation to suggested nutrition actions, as well as the status of their Operation Timbang Plus (OPT) reports and status of actions in terms of coverage of nutritionally vulnerable and disadvantaged populations - A similar assessment should be conducted annually to update the local nutrition action plan and the annual investment program - LGUs: monitor their nutrition action plans in accordance with the guidelines to be adopted and issued by the NNC - NNC: provide LGUs with templates and tools to facilitate and simplify assessment and cost of these actions; pursue efforts to strengthen the capacity of DILG Field offices to assist in LGU compliance with the law - DILG: monitor nutrition indicators through local governance performance management systems profiling, and consider inclusion in the Seal of Good Local Governance (SGLG) criteria
Newborn and Maternal Care		
Date Published: 01 December 2009 DOH Administrative Order 2009-0025 Adopting New Policies and Protocol on Essential Newborn Care	Healthcare System	- Integration of the protocol on maternal packages of PhilHealth - Integration of protocol to Basic Emergency Obstetric and Newborn Care and (BEmONC) training module - Provision of globally accepted evidence-based essential newborn care protocol for MNCHN service delivery network and referral system at community level and facility level - Defined roles and responsibilities of different DOH offices and other agencies in implementation of the newborn protocol, training, monitoring, evaluation, research, and others
	Enforcement and Monitoring	- LGU- and DOH-retained hospitals, PhilHealth, and CHD to monitor and evaluate implementation of maternal and newborn care policies, including this protocol, and to update current DOH and LGU databases to include more newborn care data. - Field Implementation and Management Office, in partnership with Disease Prevention and Control Bureau (DPCB) and Bureau of Local Health Development (BLHD), to develop monitoring and evaluation tools and conduct monitoring and evaluation on maternal and newborn care policy activities - National Epidemiology Center to recommend ways and schemes in data collection and reporting based on indicators identified by newborn care
Date Published: 13 July 2020 DOH Department Order 2020-0319 Interim Guidelines on COVID-19 Management of Pregnant Women, Women About to Give Birth, and Newborns	Healthcare System	Issuance of interim guidelines for management of probable and confirmed cases of women, newborns, infants, and young children in health facilities to prevent and control COVID-19 transmission, which includes management of infant feeding concerns during COVID-19 pandemic - Regardless of COVID-19 status, provision of lactation station, maternal nutrition counseling, and practical infant feeding support must be provided - Enforcement on Philippine Milk Code: donations of formula milk are prohibited; BMSs can only be used after all means have been exhausted
	Health workers	Should encourage and support breastfeeding
	Enforcement and Monitoring	Healthcare facilities are tasked to establish a communication channel to monitor breastfeeding status and provide alternative mechanisms to support breastfeeding at home
Maternity Leave		
Date Published: 22 July 1996 Republic Act No. 8282 Social Security Act of 1997 Amended RA 1161 for strengthened and expanded coverage and increased benefits	Maternity Leave Benefit	- Granted maternity leave benefits to female members of the Social Security System (SSS). The daily maternity benefit was 100% of the average daily salary credit for sixty (60) days for normal deliveries and 78 days for cesarean delivery. Covered only the first four deliveries or miscarriages - Compulsory coverage of workers with at least PHP 1000 monthly income
Date Published: 20 February 2019 Republic Act 11210 An Act increasing the Maternity Leave period to One Hundred Five (105) 1 days of Female Workers with an option to extend for an Additional Thirty (30) days without pay, and Granting an additional Fifteen (15) days for Solo Mothers, and for Other Purposes. Otherwise known as the “Expanded Maternity Leave Law”		- Female workers are granted 105 days maternity leave with full pay, regardless of whether the delivery was normal or cesarean, with an option to extend for an additional 30 days without pay - Maternity leave is granted for every pregnancy regardless of frequency - Provision of additional 15 days maternity leave with full pay to solo parents - Grants 60 days of maternity leave for women who suffer miscarriages or emergency termination of pregnancy

## Appendix B

**Table A2:** Summary of local ordinances related to breastfeeding promotion and maternity protection ^1^.

Policies and Provisions	Number of Issuances			Total
	Provincial	Municipal	City	
Maternity protection	3	17	23	43
Maternity leave	-	-	-	-
Maternity cash entitlement	-	4	2	6
Health protection	-	1	3	4
Lactation breaks	1	4	12	17
Lactation facility	4	12	19	35
Monitoring and enforcement	-	6	14	20
Breastfeeding promotion and protection	2	14	27	43
Monitoring and enforcement	-	2	14	16
Informational and educational materials	-	7	10	17
Promotion to general public	1	9	16	26
Promotion in healthcare facilities	1	10	16	27
Engagement with health workers and systems	1	13	20	34
Labeling	-	0	4	4
Other	7	13	17	37
Funds and support for BNSs/BHWs	1	3	10	14
Re-organization of nutrition committee or technical working group	6	10	7	23

BNSs/BHWs—barangay nutrition scholars and barangay health workers. ^1^ From the Philippines National Nutrition Council Policy Database—Compendium of Local Ordinances and Issuances on Nutrition. Available online: https://www.nnc.gov.ph/policy-database (accessed on 19 April 2022).
